# Management of Anemia of Inflammation in the Elderly

**DOI:** 10.1155/2012/563251

**Published:** 2012-10-03

**Authors:** Antonio Macciò, Clelia Madeddu

**Affiliations:** ^1^Department of Obstetrics and Gynecology, Sirai Hospital, 09013 Carbonia, Italy; ^2^Department of Medical Oncology, University of Cagliari, 09124 Cagliari, Italy

## Abstract

Anemia of any degree is recognized as a significant independent contributor to morbidity, mortality, and frailty in elderly patients. Among the broad types of anemia in the elderly a peculiar role seems to be played by the anemia associated with chronic inflammation, which remains the most complex form of anemia to treat. The origin of this nonspecific inflammation in the elderly has not yet been clarified. It seems more plausible that the oxidative stress that accompanies ageing is the real cause of chronic inflammation of the elderly and that the same oxidative stress is actually a major cause of this anemia. The erythropoietic agents have the potential to play a therapeutic role in this patient population. Despite some promising results, rHuEPO does not have a specific indication for the treatment of anemia in the elderly. Moreover, concerns about their side effects have spurred the search for alternatives. Considering the etiopathogenetic mechanisms of anemia of inflammation in the elderly population, an integrated nutritional/dietetic approach with nutraceuticals that can manipulate oxidative stress and related inflammation may prevent the onset of this anemia and its negative impact on patients' performance and quality of life.

## 1. Introduction

The number of elderly individuals is expected to reach unprecedented levels in the twenty-first century. Anemia represents an emerging global health problem that negatively impacts quality of life in a significant proportion of the elderly population and requires an ever-greater allocation of healthcare resources. 

Anemia of any degree is recognized as a significant independent contributor to morbidity, mortality, and frailty in elderly patients. Although anemia has often been considered a normal consequence of aging, the pathophysiology of such an age-related decline in erythrocyte production is obscure, and efforts to understand anemia in elderly individuals have become a major target of research interest. Recent studies suggest that, although anemia likely arises in part from the cumulative effect of age-related comorbidities and physical decline, there are still age-specific changes in the hematopoietic system that influence red blood cell production. Understanding of these changes could have important diagnostic and therapeutic implications for addressing this common problem. 

### 1.1. Incidence

The incidence and prevalence of anemia increase with advancing age [1]. A fall in hemoglobin levels occurs in the eighth decade of life and may be part of normal aging. In the third National Health and Nutrition Examination Survey (NHANES III), a nationally representative study of noninstitutionalized civilian adults in the USA, the overall prevalence of anemia among adults aged ≥65 years was 11.0% in men and 10.2% in women. In that study, anemia was defined according to WHO criteria (hemoglobin concentration ≤ 12 g/dL in women and ≤ 13 g/dL in men). The vast majority of anemia cases were mild, with less than 1% of older community-dwelling adults having hemoglobin concentrations below 10 g/dL, and less than 3% were below 11 g/dL. Interestingly, the prevalence of anemia increased significantly with age, that is, up to 26.1% in men and 20.1% in women aged 85 years and over [1].

### 1.2. Social Impact

Up until a few years ago, relatively low hemoglobin concentrations were considered a common laboratory finding in the elderly, judged by physicians as a sign without clinical relevance or as a marker of an underlying chronic disease having no independent influence on health. In recent years, several studies have challenged this widespread perception of anemia as an innocent bystander, reporting increased disability, morbidity, and mortality in the anemic elderly. An association between mortality and anemia in the elderly was observed in several major cohort studies and remained significant even after excluding older adults with comorbid conditions (e.g., cardiovascular disease, cancer, kidney disease) [2–6].

Anemia in older persons is also associated with decreased physical performance, disability in daily living, cognitive impairment, depression, a diminished quality of life, and an increased number of hospital admissions. Moreover, low hemoglobin levels have been found to be a risk factor for poor mobility, increased frailty, and decreased executive function in women, and decreased motor performance in all individuals [7]. For example, in community-dwelling women aged 70–80 years, Chaves et al. demonstrated that anemia was associated with an increased probability of self-reported mobility limitations (difficulty with walking one-quarter of a mile or climbing 10 stairs) [8]. Similarly, Penninx et al. [9] prospectively showed that anemia was associated with decreased performance in objectively measured balance, the ability to rise from a chair, and walking speed over a 4-year time period in community-dwelling adults aged 71 years and older. Furthermore, muscle strength as well as muscle mass and density measured by quantitative computed tomography were significantly lower in anemic than in non-anemic community-dwelling older adults. 

Additionally, several large high-quality population-based studies have documented anemia or low hemoglobin as predictive for dementia or cognitive decline [10, 11] Moreover, the data seem to suggest that mild anemia that develops later in life may significantly contribute to health vulnerability and poor clinical outcomes in the elderly and, in turn, be associated and induced by the same factors responsible for these events (comorbidities). Elderly individuals with an acquired disorder such as mild anemia of chronic disease demonstrate a fivefold increase in mortality risk, whereas those with an inherited, generally asymptomatic lifelong condition such as beta-thalassemia minor are not at increased risk of mortality. This hypothesis was confirmed by a large prospective population-based study performed between 2003 and 2007 in a population of 7,536 elderly (65 to 84 years old) residents in Biella, Italy. The authors found that mild anemia was prospectively associated with clinically relevant outcomes such as an increased risk of hospitalization and all-cause mortality [12].

The specific mechanisms by which anemia may adversely affect relevant health-related outcomes in the elderly are unknown. At least in theory, anemia may contribute to the decline in physical function by interfering with oxygen delivery to the brain, heart, and muscles. However, an alternative explanation is that anemia may be a consequence of the underlying comorbid diseases and frailty that cause disability. Addressing this question is critical for geriatric research. Because the prevention and management of disability is a major goal of geriatric medicine, the possibility that anemia is one of the few potentially reversible causes of disability in older persons is particularly appealing. However, there is currently little information on whether correcting mild anemia in older persons can prevent or reverse physical impairment and/or disability or increase life expectancy in patients with multiple chronic morbidities [13].

Thus, considering the steep increase in the prevalence of anemia in older individuals, and the exponential rise in the number of older individuals in our aging society, anemia in older individuals may have a relevant impact on healthcare needs and costs [14]. The adequate diagnosis and treatment of anemia in older persons seems to be therefore of vital importance. 

## 2. Etiopathogenesis of Anemia in the Elderly

To date, it is generally accepted that the causes of anemia in the elderly can be divided into three broad groups as follows: (a) nutrient-deficiency anemia, which is most often iron-deficiency anemia; (b) anemia of chronic disease, perhaps better termed anemia of chronic inflammation; (c) unexplained anemia [15]. In the NHANES III study, 34% of all cases of anemia in elderly patients were caused by folate, vitamin B12, or iron deficiency, either alone or in combination (nutrient-deficiency anemia); 20% were due to chronic diseases; in 34% the cause remained unexplained (including myelodysplasia) [16]. NHANES III classified iron-deficiency anemia with other nutritional anemias, a classification that might be correct in the developing third world, but in North America and Western Europe, iron deficiency is more often caused by blood loss and the cause must be sought and dealt with [17]. In fact, while some cases of iron deficiency results from diet, blood loss through gastrointestinal lesions is the primary cause in older adults [18].

However, anemia in the elderly may often not be attributable to a single cause because it is logical to hypothesize that various factors can participate to its pathogenesis, including the following: renal insufficiency (approximately 12% as reported by NHANES study), sex hormone deficiency, stem cell proliferative decline, and metabolic disorders. Approximately 60% of cases of nutrient-deficiency anemia are associated with iron deficiency. On the other hand, recognition of the nutritional deficiencies (and thus screening for them) is a prerequisite of successful therapy. In fact, about one-third of nutrient-deficiency anemia are usually associated with vitamin B12, most frequently related to food-cobalamin malabsorption, and/or folate deficiency and are easily treated with nutrient replacement [19, 20].

Despite all these considerations, a particular and peculiar role seems to be played by the anemia associated with chronic inflammation termed as “anemia of inflammation” (AI), which remains the most complex form of anemia to treat.

### 2.1. Anemia of Inflammation

Anemia of inflammation (AI) has historically been termed the “anemia of chronic disease” and is most commonly observed in association with infection, rheumatologic disorders, malignancy, and other chronic illnesses. On a biochemical level, it is classically characterized by low serum iron and low iron binding capacity in the setting of an elevated or normal serum ferritin. Just as important seems to be the direct role of inflammatory mediators in interfering with erythropoiesis and control of erythropoiesis exerted by the kidney. In fact, patients with chronic inflammatory diseases have been demonstrated to have decreased red cell survival, disorders of erythropoiesis, EPO levels low for the degree of anemia, and progressive EPO resistance of erythroid progenitors. The relative role and interplay of these mechanisms in the development of inflammatory anemia remain unknown, as are the potential common pathways that may link them [21]. 

Until now chronic inflammation has been considered as the main cause of these events; however, the origin of this nonspecific inflammation in the elderly has not yet clarified. It seems more plausible that the oxidative stress that accompanies the evolution of our life is the real cause of the chronic inflammatory conditions of the elderly and that the same oxidative stress is actually a major cause of this anemia. Surprisingly, due to its ability to accumulate over time, oxidative damage has emerged as a consequence of longevity *per se*. Importantly, antioxidative stress adaptations are not subjects to evolutionary pressure at postreproductive ages, which further contributes to the buildup of oxidative damage in aging individuals. 

There is generally agreed to be a correlation between aging and the accumulation of oxidatively damaged proteins, lipids, and nucleic acids. Oxidatively modified proteins have been shown to increase as a function of age. Studies reveal an age-related increase in the level of protein carbonyl content, oxidized methionine, protein hydrophobicity, and cross-linked and glycated proteins, as well as the accumulation of less active enzymes that are more susceptible to heat inactivation and proteolytic degradation. A number of age-related diseases have been shown to be associated with elevated levels of oxidatively modified proteins. The accumulation of oxidatively modified proteins may reflect deficiencies in one or more parameters of a complex functional pathway that maintains a delicate balance between the presence of a multiplicity of prooxidants, antioxidants, and repair, replacement, or elimination of biologically damaged proteins [22].

#### 2.1.1. Oxidative Stress and Inflammation of Aging

Oxidative stress is defined as an unbalanced metabolic condition between the oxidant and antioxidant systems of our body, with prevalence of the oxidants. The oxidant systems, represented mainly by reactive oxygen species (ROS), are present in the body as normal products of O_2_ energy metabolism but can be overexpressed in various disease conditions. Oxygen-free radicals are intermediate compounds derived from the univalent reduction of oxygen by electrons and protons characterized by an unpaired electron in the external orbit, which makes them highly reactive (O_2_^−^, OH^−^, H_2_O_2_). Due to this reactivity, these radicals seek out balance by taking electrons from other molecules with which they come into contact, molecules that thus become unstable and that in turn become oxidants, triggering an unstable chain mechanism. Superoxide anion and H_2_O_2_ are normally the least active but in the presence of transitional forms of some metals, such as Fe^3+^, generate the more reactive OH^−^. These compounds, normally harmless but potentially toxic, are produced continuously during the various phases of the aerobic glucose metabolism and some cells of the immune system (neutrophils and macrophages) use them as mediators of their response. The destructive action of free radicals mostly affects cells, in particular the fats that form the cellular membranes (lipoxidation), sugar phosphates, nuclear proteins, enzymes, and especially DNA, where they alter genetic information [23]. 

Due to this process, the body possesses various detoxification systems that enable the deactivation of these molecules once their aims have been achieved. These systems include antioxidants synthesized in the body and exogenous systems introduced with food. Endogenous antioxidants include both enzymatic system, such as glutathione peroxidase (GPX), catalases, and superoxide dismutase (SOD); and nonenzymatic defense systems, such as reduced glutathione (GSH), histidine, iron binding proteins (transferrin and ferritin), dihydrolipoic acid, and reduced Q10 coenzyme. Glucose, through the pentose phosphate pathway, plays a major role in the synthesis of antioxidant-reducing compounds. Reduced glutathione is the main pathway, and its role is crucial in maintaining the right balance of the reductive oxide system, thus counteracting the toxic effect of oxidative stress. Its synthesis occurs in the cell through a complex biochemical pathway composed of different and well-researched enzymatic systems. During the detoxification reaction of H_2_O_2_, GSH is oxidatively transformed into oxidized glutathione (GS-SG) by the GPX enzyme. Glutathione peroxidase is a selenium-dependent enzyme, and at low concentrations a correlation is observed between GPX and selenium state. Indeed, GPX levels provide a more accurate measure of selenium concentrations that does the direct measurement of the selenium level through atomic absorbance. The reduction of GS-SG to GSH is catalyzed by GS-SG reductase, which uses NADPH, synthesized in the pentose phosphate pathway starting from glucose, for its strong reducing power. Glucose-6 phosphate dehydrogenase (G6PD) is the first and most important enzyme of the phosphate pentose pathway and recent studies have shown that this enzyme plays a very important protective role in counteracting ROS toxic action. Moreover, NADPH is required for the formation of active tetramer catalysis that catalyzes the reduction of H_2_O_2_ to H_2_O and O^2^.

Oxidative stress is considered to be a major detrimental factor limiting longevity, as originally postulated in the free radical theory of aging. Oxidative stress leads to the accrual of damaged/misfolded proteins, an increased mutagenesis rate and inflammation. Peculiarly, due to its ability to accumulate over time (as was observed in many neurodegenerative disorders), oxidative damage also emerges as a consequence of longevity *per se*. Importantly, anti-oxidative stress adaptations are not subjects of evolutionary pressure at post reproductive age, which further contributes to the buildup of oxidative damage in aging individuals [24].

Reactive oxygen species (ROS) are constantly produced in biological tissues and play a role in various signaling pathways. Abnormally high ROS concentrations cause oxidative stress associated with tissue damage and deregulation of physiological signals. The changes in insulin function by altering the proper glucose metabolism for energy supply and antioxidant synthesis would be responsible for the dysmetabolic situations typical of the elderly characterized by a state of oxidative stress. Molecular studies have revealed that the insulin-independent basal activity of the insulin receptor is increased by ROS and downregulated by certain antioxidants. Complementary clinical studies have confirmed that supplementation of the glutathione precursor cysteine decreases insulin responsiveness in the fasted state. Indeed, aging has been suggested to be driven by overactivation of signal-transduction pathways such as the nutrient-sensing pathway, and the consequent oxidative stress may be both one of its activators and effectors. Then, the aging process is characterized by ROS-mediated mitochondrial damage, an increase in iron deposition, deregulation of Ca^++^ homeostasis, and postabsorptive plasma cysteine homeostasis, impaired autophagy, and sirtuin activity secondary to aberrant insulin receptor signaling. These altered pathways may represent pivotal mechanisms in a vicious cycle leading to progressively increasing intracellular ROS concentrations and oxidative stress [25].

In turn, ROS are able to influence inflammatory responses. Indeed, the inflammatory response initiated by the (NLRP3) inflammasome is triggered by a variety of situations of host “danger”, including infection and metabolic deregulation. Inflammasomes are intracellular multiprotein sensors, which can recognize a large set of danger signals, induced either by pathogens or cellular stress, and once activated, they subsequently stimulate inflammatory responses. There are several subfamilies of NOD-like receptors (NLR) but emerging data indicates that the NLRP subfamily, in particular the NLRP3 member, is the major sensor for “intracellular danger-associated molecular patterns” (DAMPs). In the case of NLRP3, the activated receptor interacts with the adaptor protein ASC that recruits the inflammatory caspase-1 (CASP-1) to the complex, which subsequently oligomerizes into penta- or heptameric inflammasomes [26].

How diverse “stresses” cause inflammasome activation is poorly understood. Recent data suggest that dysfunctional mitochondria generate ROS, which is required for the NLRP3 inflammasome activation. On the contrary, the NLRP3 inflammasome is negatively regulated by autophagy, which is a catabolic process that removes damaged or otherwise dysfunctional organelles, including mitochondria [27]. The mitophagy/autophagy blockade leads to the accumulation of damaged, ROS-generating mitochondria, which in turn activates the NLRP3 inflammasome. Resting NLRP3 localizes to endoplasmic reticulum structures, whereas upon inflammasome activation both NLRP3 and its adaptor ASC redistribute to the perinuclear space where they colocalize with endoplasmic reticulum and mitochondria organelle clusters. Notably, both ROS generation and inflammasome activation are suppressed when mitochondrial activity is deregulated by inhibition of the voltage-dependent anion channel. This point indicates that the NLRP3 inflammasome senses mitochondrial dysfunction and may explain the frequent association of mitochondrial damage with inflammatory diseases [28]. There is also the possibility that ROS could directly oxidize thiol groups in leucine-rich repeat (LRR) domain of NLRP3 and in that way activate the inflammasome pathway [29].

Upon activation, the NLRP3 inflammasome serves as a platform for activation of the cysteine protease caspase-1, which leads to the processing and secretion of the proinflammatory cytokines interleukin-1*β* (IL-1*β*) and IL-18 [30]. NF-*κ*B signaling is a crucial inducer of NLRP3 expression [31]. *In vivo* and *in vitro* studies indicate that the age-related increase in the steady state level of interleukin 6 (IL-6) mRNA and IL-6 production results from enhanced binding of the redox-sensitive transcription factor nuclear factor *κ*B (NF-*κ*B) to the IL-6 promoter [32]. The age-related increase in IL-6 is reminiscent of the systemic increase in the concentration of the inflammatory cytokine, TNF-*α*, which is also controlled by NF-*κ*B. Accordingly to this finding, the increase in these cytokines was ameliorated by treatment with the GSH prodrug N-acetyl cysteine (NAC) [25].

Then, dysregulated NLRP3 inflammasome activation is associated with both heritable and acquired inflammatory diseases. In addition to the processing and secretion of pro-inflammatory cytokines such as IL-1*β*, NLRP3 inflammasome activation also influences cellular metabolic pathways such as glycolysis and lipogenesis. Mapping the connections between mitochondria, metabolism, and inflammation is of great interest as malfunctioning of this network is associated with many chronic inflammatory diseases.

**Inflammaging**

In 2000, Franceschi et al. [33]. coined the term “inflammaging” in order to refer to a low-grade proinflammatory status appearing during the aging process. They emphasized the role of macrophages as well as cellular stress and genetic factors in the generation of the inflammaging condition. In addition, they hypothesized that this inflammatory environment could predispose the organism to the development of several age-related diseases. The presence of a pro-inflammatory phenotype in aged mammals is evident by (i) increased expression of genes linked to inflammation and immune responses in the tissues of old humans [34], (ii) higher level of cytokines in serum, for example, IL-6 and TNF-*α* [35], (iii) activation of NF-*κ*B signaling which is the master regulator of inflammatory responses [36].

It should be noted that different cellular stresses and the aging process could stimulate NF-*κ*B signaling and probably enhance the priming and potentiating of the inflammasome activation. It seems that NLRP3 could be a sensor for metabolic stress recognizing ROS production [37]. The increased level of ROS induced by oxidative stress was one of the first stimuli, which was demonstrated to trigger NLRP3 activation and promote CASP-1-dependent IL-1*β* secretion [38]. In turn, IL-1*β* impairs insulin signaling and promotes insulin resistance in mice.

**Figure 1:**
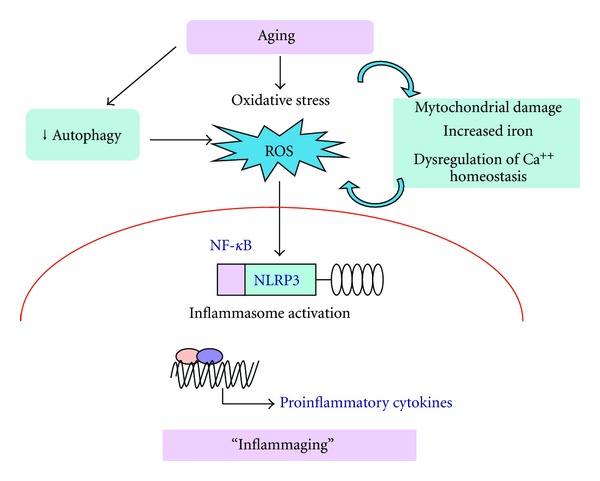
Oxidative stress and inflammation of aging. Abbreviations: ROS, reactive oxygen species; NF-*κ*B, nuclear factor *κ*B.

The aging process involves a progressive decline in cellular and organism function; in particular, defects in mitochondrial uptake and degradation could increase ROS production and stimulate inflammasomes (Figure 1). There are several regulatory mechanisms, which could augment the appearance of inflammaging phenotype during the age-related deficiency of autophagy. It is well known that increased levels of ROS can activate NF-*κ*B signaling via multiple mechanisms [39]. For instance, several redox-sensitive protein kinases and phosphatases can stimulate IKK-NF-*κ*B signaling and thus induce and maintain an elevated priming state of inflammasome system. Moreover, a decline in autophagy can stimulate the activating kinases of the NF-*κ*B complex, that is, I*κ*B kinase *α* and *β* (IKK*α*/*β*) and NF-*κ*B-inducing kinase (NIK), which is degraded via selective autophagy. Both of these responses are typical hallmarks of inflammaging and inducers of inflammasomes.

In conclusion, the decline in autophagy during aging creates problems in cellular housekeeping functions, which stimulate NF-*κ*B signaling in order to directly, or via inflammasomes, trigger an age-related pro-inflammatory phenotype. Moreover, there are indications that inflammatory signaling can repress autophagy and thus induce this destructive interplay between autophagy and inflammasomes. For instance, TNF-*α*, an inflammatory cytokine, can induce or repress autophagy in an NF-*κ*B-dependent manner. In the presence of NF-*κ*B signaling, TNF-*α* activates mTOR, a major autophagy inhibitor, whereas in cells lacking NF-*κ*B activation, TNF-*α* treatment stimulates the expression of Beclin 1, an enhancer of autophagy. Both of these responses are dependent on the TNF-*α*-induced ROS production [40].

#### 2.1.2. Chronic Inflammation and Ageing

Aging is associated with profound alterations in the innate immune system as exemplified by alterations in the T and B cell compartments, involution of the thymus gland, functional decline in the monocytes and macrophages, low expression of Toll-like receptors from activated splenic and peritoneal macrophages, and an altered secretion of several chemokines and cytokines. Moreover, aged residential phagocytes such as macrophages and neutrophils within host sometimes exhibit inappropriate respiratory burst with concomitant release of reactive nitrogen and oxygen intermediates. In sharp contrast, mitogen-activated peripheral blood mononuclear cells isolated from elderly population show higher production of pro-inflammatory cytokines, such as IL-1, IL-6, and TNF-*αex-vivo*, compared to young people. Upregulated COX-2 expression, and the resulting increase in the production of prostaglandin E_2_ (PGE_2_), has been reported as critical factor associated with age-related inflammatory changes [41].

Moreover, the prevalence of chronic inflammatory diseases increases dramatically with advancing age. In addition to cancer and arthritis, atherosclerosis, diabetes, and other common disease processes are associated with elevated serum pro-inflammatory cytokines, suggesting that inflammatory processes are common in the pathogenesis of many age-associated diseases. Furthermore, pro-inflammatory cytokine levels increase with increasing adiposity and it has been speculated that the age-associated changes in relative body composition account for much of the observed rise in these inflammatory markers with advancing age. Moreover, because both estrogen and testosterone are inhibitors of NF*κ*B activity and thereby the transcription of several inflammatory mediators, including interleukin-6, it has been suggested that those endocrine changes that occur at menopause or andropause result in a constitutively increased presence of inflammatory mediators [42, 43].

It is unclear whether chronic inflammation reflects primary age-related immune dysregulation or a systemic response to the presence of comorbid conditions. Some studies have suggested that, in normal healthy elderly individuals, the circulating levels of inflammatory cytokines are not elevated, whereas others have suggested that these markers are elevated even in the absence of comorbid disease. In the large InCHIANTI study of more than 1300 individuals, levels of IL-6, IL-1 receptor antagonist, IL-18, C-reactive protein, and fibrinogen were all elevated in patients over age 65, although when adjusted for cardiovascular risk factors and morbidity, the elevation was small [44]. Taken together, there exists some debate as to whether an assaying cytokine level in the peripheral blood is an accurate method for estimating chronic inflammatory states. Furthermore, cytokine levels may be variable and subject to transient perturbations in the setting of acute stresses and comorbidities. Therefore, studies linking cytokine promoter polymorphisms to the development of various inflammatory diseases suggest that genetic variations that determine overall inflammatory responsiveness may be better markers of disease susceptibility than peripheral cytokine levels. Consequently, the assessment of functional polymorphisms in candidate genes offers a worthwhile investigative approach to assessing clinical outcomes. 

Moreover, even if there is strong evidence that many markers of inflammation, including TNF-*α* and IL-6, are increased in the elderly population, studies detailing evidence for a chronic proinflammatory state contributing to the pathogenesis of elderly anemia are few in number and limited by small sample sizes, lack of correlation with hepcidin and EPO levels, and reliance on measurements of peripheral blood cytokine levels as determinants of the presence of a chronic inflammatory state [45]. Ferrucci et al. compared erythropoietin (EPO), inflammatory markers, and major comorbidities between older subjects with normal hemoglobin levels and those with different etiologic forms of anemia, including unexplained anemia. Participants were a representative sample of 964 persons aged ≥65 years, with no evidence of bleeding. Anemia was defined as hemoglobin <13.0 g/dL in men and 12.0 g/dL in women, and classified as a result of chronic kidney disease, iron deficiency, chronic disease and B12/folate deficiency anemia, or unexplained anemia. Participants with anemia of chronic diseases had significantly higher IL-6 and C-reactive protein (CRP) levels, while those with unexplained anemia had significantly lower CRP than nonanemic controls. Unexplained anemia was characterized by unexpectedly low EPO and low lymphocyte count. Unexplained anemia was associated with reduced kidney EPO response, low levels of pro-inflammatory markers, and low lymphocyte counts [46]. 

## 3. Oxidative Stress, Inflammation, and Erythropoiesis

### 3.1. Erythropoiesis

Knowledge of hemopoiesis, especially erythropoiesis, and of its regulatory mechanisms is crucial to understand the pathophysiology of the different forms of anemia to implement the most appropriate and effective therapeutic options. Hemopoiesis is a process through which it is possible to maintain a constant balance in the number of different elements in the blood, compensating with the production of new cells to replace those that, having completed their vital cycle, die. The speed at which the new cells are produced is modulated by various physiological requests and can be constantly modified in response to different pathological conditions. Hemopoiesis is regulated by a mechanism that involves complex interactions between cells in the bone marrow microenvironment and is controlled by several glycoprotein hormones and by peptides defined as “hemopoietic growth factors.” The growth factors can directly stimulate cell proliferation or can enhance the proliferative effect of other factors. The majority of these growth factors stimulate more than one cell line, as different cells can share some of the subunits of the same receptor localized in the cell membrane.

Growth factors can be classified according to the cell lines mostly affected by their action or according to the level of maturation of the hemopoietic process on which they act. They can thus be distinguished as follows: (a) early activators: specific for a particular cell line, they stimulate the proliferation and promote the differentiation of multipotent cells in different cell types, which will then become the progenitors of the various cell series; (b) late activators: such as erythropoietin, this acts in a specific way on cell lines already programmed towards a set type, inducing their multiplication/proliferation and maturation.

Erythrocytes are derived from an undifferentiated progenitor, the pluripotent stem cell, found in the hemopoietic marrow, which, like all stem cells, is able to replicate itself and differentiate into more mature cells. The most undifferentiated progenitor programmed in an erythroid manner, isolated both from the marrow and the peripheral blood, is the “burst forming unit erythroid” (BFU-e). The BFU-e is mostly quiescent, able to migrate, has a high proliferative capacity and for its replication and survival requires a specific linear or multilinear lymphomonocyte growth factors such as interleukin (IL)-3, IL-4, IL-9, IL-12, granulocyte-macrophage colony-stimulating factor, (GM-CSF) and stem cell factor, SCF, in addition to the erythroid line specific growth factor, that is, EPO. The BFU-e has a reduced number of EPO receptors, which are both mitogens and survival factors. After 10–15 days of appropriate stimulation, the BFU-e produces huge cells with morphological characteristics typical of the erythroid series precursors. These more mature BFU-e cells are called colony-forming units erythroid (CFU-e). The CFU is thus the direct descendant of the BFU-e, it has a limited proliferative potential, is not able to self-regenerate, and has an absolute need for EPO in at least 2 of its 6 divisions, and after 4–7 days of stimulation forms smaller colonies of erythroid cells that are very sensitive to EPO [47]. The specifically stimulated CFU-e thus gives rise to the first cell morphologically recognizable as an erythroid in the bone marrow, that is, the proerythroblast. This cell subsequently matures through the stages of basophilic, polychromatophilic, and orthochromatic erythroblasts, finally resulting in reticulocytes. More precisely, the transformation into a mature erythroblast requires a period of approximately 4 days. During this time, the nucleus becomes smaller and Hb is synthesized in the cytoplasm; after the last mitosis, the nucleus, very small in size by now, is eliminated by the proerythroblast, which thus transforms into reticulocytes. This cell, in turn, remains in the marrow for a further 2-3 days prior to entering the circulation where, after approximately 24 hours, after losing its mitochondria and ribosome, it resembles a mature erythrocyte. Erythropoiesis is thus the result of the following 4 distinct processes:

- proliferative capacity of the erythroid progenitor pool in the bone marrow;
- intensity of the stimulus to the erythrocyte synthesis;
- nutrient availability (iron is the most important);
- erythrocyte survival (which can be reduced due to hemorrhage or early erythrocyte destruction).

Erythropoiesis is a hierarchical process by which the erythrocyte mass is maintained at a constant level according to a dynamic balance whereby erythrocytes lost due to senescence are replaced by an equal quantity of new erythrocytes. The main function of erythrocytes is to transport oxygen (O_2_) from the lungs to the other tissues and transport carbon dioxide (CO_2_) in the opposite direction. As the basal consumption of oxygen is 4 mL/kg/min and the overall body oxygen stores are only 20 mL/kg, it is crucial to maintain an adequate and stable erythrocyte mass. This mass should also be capable of expanding in response to tissue hypoxia due to different causes.

Erythropoiesis is thus a continuous and orderly process by which progenitor cells that have been programmed in an erythrocyte manner proliferate and differentiate themselves into mature erythrocytes to maintain stable or expand the erythrocyte mass. The final aim is the production of an adequate store of long-lived erythrocytes with a mean cell volume (MCV) of 90 femtoliters (range 80–100) and a median corpuscular hemoglobin concentration (MCHC) of 34 g/dL (range: 32–36). The two processes of proliferation and differentiation should be balanced as the transport of O_2_ is the main function of erythrocytes, and MCHC is the erythropoiesis-determining factor. If iron stores or the capacity to synthesize Hb are reduced, the erythrocyte progenitors will sacrifice their cell volume in order to maintain MCHC within normal levels, and the number of subdivisions, usually limited as erythrocytes progenitors have differentiated in a terminal manner, will increase to achieve this objective. In this way, proliferation overcomes differentiation.

### 3.2. Erythropoietin

The great majority of erythropoietic growth factors is produced constitutively and acts locally; many are ligands and others, although soluble, are limited in their capability for action by glycosaminoglycan link with the extracellular matrix of the bone marrow where they are produced. EPO is the main erythropoietic growth factor and behaves like a hormone as it is produced in the kidney and liver and acts on the erythroid progenitors in the bone marrow. EPO matches the production quantity of erythrocytes to the O_2_ request at the tissue level through a refined and complex O_2_-sensitive system mediated by the hypoxia-inducible factor 1 (HIF1). With the onset of tissue hypoxia, HIF1 enters the cell nucleus within 2 minutes, dimerizes with itself, and promotes the transcription of the EPO gene. This step is followed by a logarithmic increase in EPO plasma concentration that is inversely proportional to Hb level and hematrocrit (Hct), with the rule being that until Hb falls below 10 g/dL and the hematocrit below 3%, EPO plasma levels do not increase beyond the normal range. Indeed, EPO has been well established as the substance that transfers the message to the erythropoietic marrow as to how much it should increase the production of new erythrocytes to guarantee an appropriate tissue oxygenation [48]. 

However, EPO production has been shown to be directly stimulated by growth factors and hormones. For instance, growth factor hormone (GH) and certain androgens and thyroid hormones can affect erythropoiesis through the activation and modulation of EPO production. Proinflammatory cytokines, the plasma concentrations of which are significantly higher in patients with chronic inflammatory diseases, can also affect EPO production. In particular, IL-1*α*, IL-1*β*, TNF*α*, and TGF-*β* inhibit the production of EPO and change the response of the erythroid progenitor cells to this irreplaceable growth factor. 

#### 3.2.1. Erythropoietin Mechanism of Action

EPO is known to act on the erythroid progenitors interacting with specific high- and low-affinity receptors localized on the cell membrane. Although these membrane receptors have not yet been well characterized, they are expressed almost exclusively on the erythroid progenitors and, in particular, on the more mature cell types, such as CFU-e and erythroblasts, although BFU-e is also somewhat stimulated. Thanks to the presence of this complex EPO receptor, the erythroid progenitors are stimulated to differentiate into mature erythrocytes, whereas when EPO is not present they face death by apoptosis. Mature erythrocytes do not have the receptor for EPO, and thus their function is not affected. Once EPO interacts with its receptors, it is rapidly internalized and metabolized until the same target cells eventually degrade it. The primary physiologic erythropoietin functions are the following: to recruit and commit immature progenitors, to guarantee the survival of erythroid progenitors preventing apoptosis, and to stimulate their differentiation and maturation into erythrocytes [49].

#### 3.2.2. Erythropoietin Physiology

EPO production is regulated at the transcriptional level, and hypoxia is the only physiologic regulatory factor of EPO gene expression. The erythroid progenitors are the main targets of EPO, both due to their high number and their high density of receptors. Similar to other erythrocyte growth factors, EPO is metabolized by its target cells following receptor binding and its internalization. No preformed EPO stores exist, and an increase in plasma levels is due only to its renewed entrance into the circulation once it is synthesized again. EPO synthesis in adult humans mainly occurs in the kidneys, specifically in the peritubular interstitial cells, with only a small proportion, usually not more than 10%, produced by the liver. The threshold for hypoxia-induced EPO liver production is very high, and although the liver is bigger in size, it is not able to compensate for an inadequate EPO production by the kidneys unless under severe hypoxic conditions.

Kidney EPO production is stimulated by tissue hypoxia through a feedback mechanism. When O_2_ kidney sensors detect the presence of hypoxic condition due to anemia, ventilation, or perfusion defects; alterations to the hemoglobin-oxygen disassociation curve; reduction of blood flow or decreased O_2_ tension in the environment, peritubular interstitial cells are stimulated to produce EPO. When the erythrocyte mass increases and the O_2_ transport increases, EPO production reverts back to normal. Interestingly, hypoxia stimulates the secretion of a high quantity of O_2_ in a short time, with the plasma concentration peak normally not lasting very long and maintained only if the conditions of extreme hypoxia persist. As soon as other compensatory mechanisms are activated, such as the increase in lung ventilation or cardiac frequency or the redistribution of blood flow, the production of EPO rapidly decreases. However, despite this reduction, the increase in the erythrocyte mass continues. This phenomenon means that significant concentrations of EPO are required for the initial activation of erythroid precursors, whereas lower concentrations are sufficient for the continuation of the differentiation and maturation processes [50]. Goldwasser and Sherwood [51] have devised an accurate radioimmunological method to determine the concentration of this growth factor in the plasma, allowing for the definition of its values in normal subjects and in different pathological conditions. Endogenous EPO dosages are measured in units, with one international unity (IU) of endogenous EPO defined as the activity that induces the same erythropoietic effect of one *μ*mol of cobalt. 

### 3.3. Erythropoiesis and Aging

Age-related changes in erythropoiesis can broadly be classified into the following two general mechanistic categories: (1) alterations intrinsic to erythroid progenitor or hematopoietic stem cells and/or the local hematopoietic microenvironment, and (2) alterations in humoral control mechanisms, particularly related to secretion of the hormone erythropoietin and possible deterioration of hypoxia-sensing mechanisms, but also due to other changes in the endocrine milieu [52].

#### 3.3.1. Inflammation, Oxidative Stress, and Impaired Erythropoiesis

**Figure 2:**
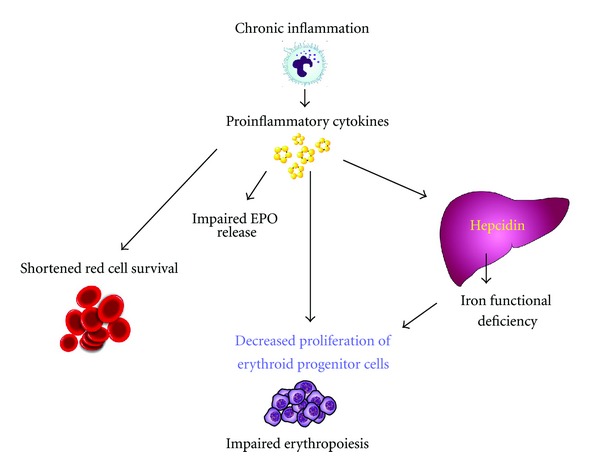
Pathogenesis of anemia of inflammation in the elderly. Abbreviations: EPO, erythropoietin.

The mechanism whereby the process of inflammation, and in particular proinflammatory cytokines, produces anemia is incompletely understood. Inflammation-associated hypoproliferative anemia has much overlap with iron deficiency, but typically serum iron is low or normal whilst ferritin is high. Based on the physiology of erythropoiesis and the results of preclinical and clinical studies, researchers have suggested the following four main mechanisms by which inflammation may affect anemia: (1) inflammation renders erythropoiesis ineffective by inhibiting the proliferation and differentiation of erythroid precursors and/or the downregulation of the biological response to EPO (EPO resistance), perhaps by downregulating the EPO receptor; (2) inflammation reduces the amount of EPO production that is normally determined by anemia-induced relative hypoxia; (3) inflammation causes the upregulation of hepcidin synthesis that, by enhancing the proteolysis of ferroportin, reduces the intestinal absorption and recycling of iron and induces iron restriction; (4) inflammation negatively affects erythrocyte survival, which is not fully compensated for by increased erythropoiesis [53] (Figure 2). 

The involvement of cytokines in the pathogenesis of late-life anemia is suggested by a number of experimental observations. T cells from poor responders to erythropoietin therapy were found to produce increased interferon gamma and tumor necrosis factor alpha (TNF-*α*) when compared with those patients with excellent erythroid response to treatment or to normal controls. Furthermore, bone marrow cell cultures treated with serum from patients with inflammation exhibited suppression of erythroid colony-forming units (CFU-e) and this effect was reversed by using antibodies against TNF*α* and or interferon gamma (IFN*γ*). Although by no means proven, it is quite possible that the presence of even mildly elevated IL-6 present on the basis of age alone, body composition change, or smoldering inflammatory disease results in inhibition of erythropoietin production and/or activation of hepcidin, both of which would result in anemia [54].

Evidences for TNF-*α* inhibiting effect on erythroid differentiation have been described 30 years ago. In 1987 Blick et al. observed a decrease in hemoglobin synthesis in cancer patients treated with TNF-*α* [55] while *in vitro* study showed that TNF-*α* inhibited the formation of BFU-e cells [56]. Later, Xiao et al. reported that TNF*α* inhibited the glycophorin A+ cells in correlation with an inhibition of erythropoiesis [57]. Moreover, an increasing hemoglobin level has been observed in patients suffering from anemia of chronic disease after an anti-TNF treatment [58]. Few publications describe the molecular mechanisms implicated and they clearly show the role of TNFR1 and NF-*κ*B, as well as other specific transcription factors, namely GATA-1 and FOG1, NF-E2, and GATA-2 [59].

Proinflammatory cytokines are also able to inhibit the proliferation and differentiation of erythroid (BFU-e and CFU-e) progenitors and to exert a direct toxic action on erythroid progenitors through the induction of ROS production by marrow stroma macrophages. Proinflammatory cytokines are also involved in the complex mechanism regulating the EPO synthesis in response to hypoxic stimulus. Patients with inflammation, infections, or cancer have a reduced response to tissue hypoxia, and EPO plasma levels are always inappropriately lower for anemia. The presence of an inflammatory response with high levels of lymphocyte-macrophage cytokines is the common denominator in these three conditions. 

Studies *in vivo* on murine models have shown that the injection of lypopolisaccharide determines a reduction of EPO synthesis and circulating levels [60]. Moreover, the responsiveness of erythroid progenitors to EPO is conversely correlated to the severity of chronic inflammation; the higher the level of IFN-*γ* e TNF-*α*, the greater is the quantity of EPO needed to allow the synthesis of CFUe [61]. Proinflammatory cytokines also negatively affect the signal transduction mechanism subsequent to EPO binding to its receptor, such as tyrosine kinase phosphorylation and the activation of mitosis [62].

The inhibitory action exerted by proinflammatory cytokines on the production of EPO [60] is at least in part due to ROS production. They damage kidney cells responsible for EPO synthesis and change the binding affinity of transcriptional factors involved in its synthesis at nuclear level.

ROS are capable of inhibiting the production of EPO from kidney tissue. *In vivo* data support the idea that reactive O_2_ species, especially H_2_O_2_, are part of the signaling chain of the cellular O_2_-sensing mechanism regulating the renal synthesis of EPO [63]. Under normoxic conditions, the iron chelator desferrioxamine and the antioxidant vitamin A increased renal Epo production, mimicking hypoxic induction. In contrast, supplementation of the perfusion medium of hypoxically perfused kidneys with the prooxidant compounds H_2_O_2_ or pyrogallol caused a significant reduction of EPO synthesis. The inhibition of EPO formation by reactive O_2_ species could be completely antagonized by desferrioxamine and the hydroxyl radical- (OH^•^)-scavenger tetramethylthiourea. Vitamin A also antagonized the H_2_O_2_-dependent inhibition of hypoxically induced Epo synthesis. Interestingly, the addition of the antioxidant vitamin A to hypoxically perfused kidneys also induced Epo production significantly.

Furthermore, cytokines and ROS, by inducing the erythrophagocytosis process, reduce circulation survival of erythrocytes. Suicidal death of erythrocytes (eryptosis) is characterized by cell shrinkage, membrane blebbing, activation of proteases, and phosphatidylserine exposure at the outer membrane leaflet. Exposed phosphatidylserine is recognized by macrophages that engulf and degrade the affected cells. Eryptosis is triggered by erythrocyte injury after several stressors, including oxidative stress. Besides caspase activation after oxidative stress, two signaling pathways converge to trigger eryptosis as follows: (a) formation of prostaglandin E (2) leads to activation of Ca (2+)-permeable cation channels, and (b) the phospholipase A (2)-mediated release of platelet-activating factor activates a sphingomyelinase, leading to formation of ceramide. Increased cytosolic Ca (2+) activity and enhanced ceramide levels lead to membrane scrambling with subsequent phosphatidylserine exposure. Moreover, Ca (2+) activates Ca (2+)-sensitive K (2+) channels, leading to cellular KCl loss and cell shrinkage. In addition, Ca (2+) stimulates the protease calpain, resulting in degradation of the cytoskeleton. Eryptosis may be a mechanism of defective erythrocytes to escape hemolysis. Conversely, excessive eryptosis favors the development of anemia. Conditions with excessive eryptosis include iron deficiency, lead or mercury intoxication, sickle cell anemia, thalassemia, glucose-6-phosphate dehydrogenase deficiency, malaria, and infection with hemolysin-forming pathogen. Eryptosis is inhibited by erythropoietin, which thus extends the life span of circulating erythrocytes [64]. Oxidative stress *per se *increases the fragility of red blood cells and decreases the rate of erythroid maturation as well as erythrocyte lifespan. *Vice versa*, serum selenium and carotenoids could theoretically protect erythrocytes from increased variation of volume of red blood cells by protecting erythrocytes from oxidative damage. Beta-carotene supplementation has been shown to protect erythrocytes from the increased osmotic fragility that is found in zinc-deficient rats. Glutathione peroxidase protects erythrocytes from oxidative damage, and in humans, selenium supplementation has been shown to increase glutathione peroxidase activities in erythrocytes [65]. 

Also energy metabolism and body composition, which are often altered in elderly as a consequence of aging and of age-related comorbidities, may influence erythropoiesis. Takeda et al. [66] hypothesized that body composition, probably through leptin action, may affect erythropoiesis and demonstrated that BMI and leptin were inversely correlated with the recombinant human EPO (rHuEPO) dose required in patients receiving hemodialysis. Moreover, low leptin levels have been associated with impaired EPO responsiveness of erythroid progenitors and the syndrome of frailty in the elderly population. Leptin—an adipokine associated with inflammation, body fat mass, and energy metabolism—has also been recently shown to induce hepcidin via JAK2/STAT3 signaling, potentially linking nutritional status to inflammation and iron homeostasis. Moreover, *in vitro* studies have suggested that leptin plays a role in enhancing erythropoiesis [67], but, certainly, this hypothesis needs more definitive analysis.

## 4. Iron Metabolism

In healthy humans, the concentration of iron in plasma and extracellular fluid is maintained at a relatively narrow range of 10–30 *μ*M, ensuring that adequate iron is available for essential cellular functions without incurring iron toxicity. The plasma iron concentration is controlled by the hepatic peptide hormone hepcidin, which regulates the major iron flows into plasma: dietary iron absorption in the duodenum (1-2 mg/d), iron recycling from senescent erythrocytes (20 mg/d), and the recovery of iron from storage in hepatocytes and macrophages (a few milligrams per day depending on iron needs). Transferrin-bound iron exits the plasma compartment destined predominantly for the bone marrow erythrocyte precursors, where it is incorporated into heme and hemoglobin. Smaller amounts of iron are taken up by other cells, where they are incorporated into myoglobin, redox enzymes, and other iron-containing proteins [68]. 

Hepcidin is a 25-amino acid peptide synthesized in hepatocytes as a larger inactive preprohepcidin composed of a signal peptide and the 60-amino acid prohepcidin. Prohepcidin is then cleaved by the prohormone convertase furin to generate mature hepcidin. The hepcidin structure is a 4-disulfide-cross-linked beta-hairpin with an N-terminal arm that is highly conserved and essential for activity. The sole known molecular target of hepcidin is the protein ferroportin, which functions as a transmembrane conduit for the transfer of cellular iron to plasma. Most cells contain very little ferroportin and do not export iron, using it only for their own metabolic needs. The professional iron exporters, including macrophages, duodenal enterocytes, hepatocytes, and placental syncytiotrophoblasts, express ferroportin and provide iron for the entire organism. The binding of hepcidin to ferroportin on the membranes of iron-exporting cells induces the endocytosis and proteolysis of ferroportin and thereby decreases the delivery of iron to plasma. The cellular uptake of iron in its various forms (dietary elemental iron and heme for enterocytes, diferric-transferrin, heme-hemopexin, hemoglobin-haptoglobin, and senescent erythrocytes for macrophages) is also subject to regulation, but the regulation of ferroportin expression on the cell membrane appears to be the predominant mode through which iron transport into plasma is controlled [69].

As would be expected of an iron-regulatory hormone, the production of hepcidin is homeostatically regulated by plasma iron concentrations and iron stores, predominantly through a transcriptional mechanism. Increased hepcidin release in response to increased iron concentrations generates a negative feedback loop that limits iron absorption and retains iron stores. The regulatory mechanism centers on a bone morphogenetic protein receptor (BMPR) and its SMAD signaling pathway that regulates hepcidin transcription. The canonical pathway, which has other important roles in development and tissue remodeling, is adapted for iron regulation by its interaction with proteins specialized in iron sensing or iron-related signaling (such as BMP6, hemojuvelin, neogenin, etc.) [70].

Typical of the anemia related to chronic inflammatory diseases is iron homeostasis changes, with iron being retained in the reticulo-endothelial macrophages. This determines an iron shift from the circulating to depot stores, a limited availability of erythroid progenitors and, subsequently, a reduction in erythropoiesis. Iron stores in the bone marrow are thus normal or even increased as are ferritin levels and iron-binding capacity, whereas sideremia can be either normal or decreased. Administration of proinflammatory cytokines (IL-1 and TNF-*α*) in mice identifies cases of anemia associated with hyposideremia [71]. This combination of events has been attributed to the increased synthesis of ferritin by macrophages and hepatocytes, induced by cytokines themselves [72]. IL-10 can also stimulate ferritin expression and induce the macrophage acquisition of iron-transferrin [73]. In chronic inflammation iron entrance into the macrophages occurs mainly through the erythrophagocytosis, and its transmembrane transport is modulated both by the “divalent metal transporter 1” (DMT1) and natural resistance-associated macrophage protein 1 (Nramp1) proteins [74, 75]. Interferon g and TNF*α* induce DMTI overexpression, thus facilitating iron entrance in the activated macrophages. Moreover, proinflammatory stimuli reduce ferroportin expression, blocking iron export from cells and thus increasing its storage [76]. Ferroportin is a transmembrane protein that exports iron from cells. This process is physiologically responsible for iron absorption by duodenal enterocytes in the blood circulation as well as for normal iron recycling and iron mobilization from hepatocytes.

 The identification of hepcidin, a protein produced by the liver and involved in the regulation of iron metabolism, has enabled a better understanding of the existing correlation between the immune system, iron homeostasis, and anemia in chronic inflammatory diseases. Infection and inflammation generate signals that dramatically increase hepcidin synthesis and release, resulting in the characteristic hypoferremia, restriction of iron flow to erythropoiesis, and the anemia of inflammation. Hepcidin transcription is particularly responsive to stimulation by IL-6, an inflammatory cytokine that activates the JAK-STAT pathway. After phosphorylation by JAK2, the STAT3 transcription factor binds to cognate motifs in the hepcidin promoter and increases hepcidin transcription [77]. Other inflammatory cytokines and microbial products may also activate hepcidin transcription. 

In detail, IL-6 was shown to induce hepcidin promoter activity in hepatocytes through STAT3 binding to the promoter. In macrophages hepcidin expression is induced through STAT1 and Toll-like receptors (TLR2 and TLR4) different from those involved in hepatocytes [78]. Hepcidin induction by IL-6 occurs within a few hours, resulting in rapid and irreversible limited iron availability for heme biosynthesis in erythroid progenitors to inhibit their proliferation. On the contrary, inflammation in mice with a hepcidin deficit does not induce hyposideremia [79].

This finding suggests that hepcidin is strongly involved in iron restriction through reduced absorbance at the duodenal level and the blockage of its release by macrophages, thus leading to a rapid drop in plasma iron levels, iron-restricted erythropoiesis, and anemia [80]. Accordingly, transgenic mice overexpressing hepcidin and mice receiving synthetic hepcidin develop mild-to-moderate microcytic, hypochromic anemia.

 As a result, hepcidin is considered to be the main mediator of anemia of inflammation, also known as anemia of chronic disease, which is commonly found in patients with chronic infections or with inflammatory disorders, such as rheumatoid arthritis, inflammatory bowel disease, cancer, and chronic kidney disease. A preliminary analysis in the InChianti study (a population-based study of older persons in Tuscany, Italy) was unable to demonstrate higher urinary hepcidin levels in older individuals with anemia of inflammation, even if it is important to point out that hepcidin levels were inappropriately high for the degree of anemia [44]. This hypothesis should still be tested in other population-based prospective follow-up studies, preferably using the serum hepcidin assays that have recently become available. Depending on the outcomes of these additional studies, future diagnostic algorithms for anemia may incorporate markers of inflammation such as CRP or even hepcidin to discriminate between classic iron-deficiency anemia (low hepcidin levels) and iron-deficiency anemia in the context of anemia of inflammation or chronic disease (elevated hepcidin levels). The results of these studies may also lead to innovative clinical trials, such as treating older patients with anemia of inflammation with anti-inflammatory agents or hepcidin antagonists such as agents that inhibit hepcidin production (e.g., anti-interleukin 6 receptor antibodies), hepcidin-neutralizing antibodies, targets against hepcidin binding site of ferroportin, or agents that inhibit ferroportin internalization [81].

## 5. Treatment of Anemia of Chronic Inflammation in the Elderly

Although the role of chronic inflammation in the etiopathogenesis of some anemias in elderly subjects has been clearly determined, a safe and appropriate treatment protocol has not yet been described. Treatment considerations for elderly patients with anemia include correcting the underlying condition, transfusing red blood cells, antagonizing of etiopathogenetic mechanisms (inducing this particular anemia), and administering of recombinant human erythropoietin (rHuEPO) (Table 1). The only experiences with a good chance of success were those made with recombinant human erythropoietin.

### 5.1. Erythropoiesis Stimulating Agents (ESAs)

Theoretically, rHuEPO may correct the endogenous erythropoietin deficiency that is likely to occur in older patients. Moreover, apart from the direct effect on erythroid precursors, ESAs have been reported to suppress hepcidin production both in animal models and in humans, through a yet unknown mechanism [82, 83].

Ershler et al. [84] completed a feasibility study using rHuEPO in ambulatory subjects with noniron deficiency anemia and inappropriately low endogenous serum erythropoietin levels. The mean baseline Hb level was 10.0 g/dL; patients were treated with 20,000 units of epoetin alfa subcutaneously weekly over 4 weeks, and the Hb was monitored every 1 to 2 weeks. The mean Hb increase was 3.5 mg/dL. All patients responded to treatment with a brisk rise in Hb concentration without unwanted effects, and most patients reported increased activity and overall feeling of well-being.

Recently, Agnihotri et al. reported the efficacy of EPO administration in elderly anemia patients who were enrolled in a randomized, controlled trial of epoetin alpha or placebo. Treated patients had a rise in hemoglobin of 2 g/dL that was associated with a reduction in fatigue and improvement in other quality of life indicators [85]. Despite the promising results of these rare and individual experiences, rHuEPO does not have a specific indication for the treatment of anemia in the elderly currently. Although these modalities are often effective, as above reported, concerns about their side effects have spurred the search for alternatives. Adverse effects during treatment with EPO are not uncommon, such as an increased incidence of thrombotic vascular effects. In addition, the use of EPO in patients with hypertension must proceed with caution, since both acute and long-term administration of EPO can significantly elevate mean arterial pressure [86]. However, it is to be noted that the increased rate of death and cardiovascular events observed in randomized trials on the use of erythropoiesis-stimulating agents in persons with anemia of chronic kidney disease or cancer has been found when hemoglobin level increased to a target above 12 g/dL or 13 g/dL [87, 88].

The potential progression of cancer has been another significant concern raised with EPO administration [89]. However, the deployments of further large-scale prospective trials that can more clearly examine the attributes and contraindications for EPO, especially in patients with neoplastic disease, are required.

The incidence of side effects, however, remains highly uncertain. Specifically, the biological background underlying the prothrombotic effects of ESAs is multifaceted (polycythemia/hyperviscosity syndrome, hypertension, thrombocytosis, platelet hyperactivity, activation of blood coagulation) and context dependent, and it most likely requires the presence of additional prothrombotic factors. Nevertheless, this clinical and biological evidence supports the hypothesis that therapy with ESAs might not be ultimately beneficial or advantageous in patients with anemia of chronic disorders, and these drugs should not be routinely used as an alternative to blood transfusion unless future studies affirm safety and clinical benefits within these populations [90–93].

### 5.2. Oral or IV Iron

Iron supplementation is recommended in patients with ferritin concentrations of <30 ng/mL and TSAT values of <15% (absolute iron deficiency). For iron deficiency anemia, the usual replacement dose is ferrous sulfate, 325 mg (65 mg of elemental iron) per day, or ferrous gluconate, 325 mg (38 mg of elemental iron) per day. However, oral iron is poorly absorbed and is commonly associated with gastrointestinal adverse effects and poor rates of patient adherence. Low-dose iron therapy, with 15 mg of elemental iron per day as liquid ferrous gluconate, effectively corrects hemoglobin and ferritin concentrations with fewer gastrointestinal adverse effects than higher iron doses. Treatment is usually continued for six months to replete iron stores. For persons who fail to respond to oral iron therapy, parenteral treatment with iron dextran or iron sucrose is usually therapeutic [94].

In patients with anemia of inflammation and functional iron deficiency, the use of IV iron supplementation is recommended due to its superior efficacy compared to oral iron. For patients without functional or absolute iron deficiency before the initiation of ESAs, the possibility that functional iron deficiency occurred during ESA therapy should be considered if hemoglobin levels do not increase after four weeks of therapy, and appropriate treatment administered. For patients treated with ESAs who have functional iron deficiency, i.v. iron is recommended as first-line treatment [95].

In fact, i.v. iron has been demonstrated to improve hemoglobin response to ESAs both in cancer [96] as well as in dialysis patients [97]. 

An alternative way to modulate iron homeostasis may be represented by lactoferrin, which is a 78-kD cationic protein present in mammalian milk (where it was originally identified, hence the name), certain mucosal secretions, and polymorphonuclear (PMN) leukocytes playing an important role in host defense against infection and excessive inflammation [98]. In a recent paper carried out in a population of 148 advanced cancer patients undergoing chemotherapy we showed similar efficacy for oral lactoferrin in comparison to i.v. iron, combined with rHuEPO, for the treatment of cancer-related anemia [99]. Moreover, the use of an orally administered compound gives further unquestionable advantages both in terms of patient compliance, because there is no need for hospitalization, and also in terms of cost savings. Indeed, it is well known that i.v. iron administration, aside from the general anaphylactic risks, also requires specifically trained medical staff and therapeutic support centers.

### 5.3. Hepcidin Antagonism

Hepcidin antagonists, agents that decrease hepcidin production or interfere with its effect on ferroportin, are currently under investigation to relieve hepcidin-mediated iron restriction and to release more iron for erythropoiesis. In theory, hepcidin antagonists can neutralize hepcidin-stimulating cytokines (e.g., BMPs and IL-6), target cytokine signaling pathways (e.g., STAT3 and BMPR-SMAD), stimulate erythropoiesis, bind and neutralize the hepcidin peptide (e.g., antibodies and other binding molecules), prevent hepcidin binding to ferroportin, or interfere with ferroportin internalization pathways. Mouse models have provided a proof of concept of the potential benefit of hepcidin antagonists, several have reached the preclinical stage and a few have reached human trials for iron-restrictive anemia [70]. The BMP pathway is at the core of the transcriptional regulation of hepcidin synthesis by iron. Various sulfated proteoglycans are known to bind BMPs, and glycosaminoglycan heparin was recently shown to decrease hepcidin concentrations in mice and humans treated for a venous thrombosis [100]. Other natural and modified natural BMP antagonists have been implicated in the hepcidin-lowering activity in vitro and/or in mouse models [101]. Whether further development of anti-BMP agents can achieve sufficient activity and specificity for iron-related signaling and treat anemia remains to be discovered.

Agents that block the hepcidin-binding site on ferroportin without inducing its internalization are also under development. Fung et al. developed a high-content screen using cells expressing the ferroportin-GFP fusion protein to identify small molecules acting as hepcidin antagonists [102]. The authors identified compounds that allow continuous iron export from cells in the presence of hepcidin. The small-molecule hepcidin antagonists include thiol modifiers that inhibit hepcidin's binding to ferroportin.

### 5.4. Antagonists of the IL-6 Pathway

Anti-IL-6 agents were originally developed for other indications but appear to have robust hepcidin-lowering effects. In a recent phase II clinical trial on the anti-human IL-6 monoclonal antibody siltuximab in women with recurrent ovarian cancer, Coward et al. [103], apart from showing that siltuximab given as a single agent has some clinical activity in recurrent, platinum-resistant ovarian cancer, demonstrated that a significant increase in Hb levels occurred in the majority of patients using siltuximab. 

### 5.5. Activators of Autophagy

As above reported activation of autophagy counteracts inflammation and related diseases. There are two potential groups of compounds that could activate autophagy, mTOR inhibitors and AMPK activators. mTOR is a key protein kinase that couples nutritional and growth factor signaling with protein synthesis, transcription and critical responses in cellular growth, proliferation, and survival. mTOR is the major inhibitor of autophagy and is involved in aging [104]. Recently, Anisimov et al. [105] demonstrated that the lifelong administration of rapamycin, a macrolide antibiotic and powerful immunosuppressant with specific mTOR-inhibiting activity, extended the lifespan in inbred female mice. Majumder et al. [106] demonstrated that the lifelong administration of rapamycin improved the spatial learning and memory performance in aging mice. Interestingly, this was associated with a decrease in the brain level of IL-1*β* but not TNF-*α*, which could be interpreted to imply that the inflammasome activity had been reduced by the rapamycin therapy. 

The utilization of AMPK activators is another strategy to stimulate autophagy for therapeutic purposes. AMPK is an evolutionary conserved sensor for disturbances in cellular energy balance and a major inducer of autophagy [107]. Recently, it has been reported that the integrated signaling network involving AMPK regulates the aging process [108]. Various studies indicate that the aging process impairs the activation capacity of AMPK signaling and thereby disturbs autophagic activity, evokes oxidative stress, and triggers the activation of inflammasomes. There are several pharmacological activators of AMPK, including AICAR (5-aminoimidazole-4-carboxamide ribonucleoside) and the clinically used antidiabetic drug metformin. Several natural products, such as berberine, curcumin, and quercetin, have been reported to activate AMPK signaling [109]. 

### 5.6. Nutraceuticals

Since time immemorial, man has used plant extracts to protect himself against several diseases and also to improve his health and lifestyle. Traditional medicines have long provided front-line pharmacotherapy for many millions of people worldwide. Medicinal extracts are a rich source of therapeutic leads for the pharmaceutical industry. The use of medicinal plant therapies to treat chronic illness, including aging anemia is thus widespread and increasing. Recently, nutritional approaches have been sought more frequently to counteract both anemia and immunological dysfunction (i.e., immunosenescence) commonly found in older subjects. In fact, many aromatic, medicinal, and spice plants contain compounds that possess confirmed strong antioxidant and anti-inflammatory components that can counteract the etiopathogenetic mechanisms that induce aging anemia. No doubt, plants are serving several purposes in health, nutrition, beauty, or medicine. With technique development and recent research, it has been proved that certain nonnutritive chemicals in plants, such as terpenoids and flavonoids that were earlier thought to be of no importance to the human diet, possess antioxidant properties. In fact, the plants are susceptible to damage by active oxygen and thus develop numerous antioxidant defenses and potent antioxidants. Recent research has proved that these can also be used as antioxidants to protect our body from various chronic diseases, such as anemia, that can weaken the immune system of the body [110]. 

Until now, no set definition of antioxidants has existed. In simple words, “antioxidants” are a type of complex compounds found in our diet that act as a protective shield for our body against certain diseases related to oxidative stress. Oxidative damage is a significant causative factor in the development of certain human diseases, aging and related problems, and antioxidants are capable of preventing or ameliorating these processes [111]. The antioxidants are classified into the following different categories:

**Enzymatic and Nonenzymatic Endogenous Antioxidants**

These antioxidants are found both in extracellular and intracellular environment and are tactically arranged within the cell to provide maximum protection against free radicals.

**Exogenous Antioxidants Derived from Natural and Dietary Sources**

Plants develop several antioxidants that aid in the antioxidant defense system, protecting plants against damage caused by active O_2_ formed by ultraviolet exposure. Certain seaweeds also function as antioxidants. Our daily diet contains vegetables, fruits, tea, wine, and other things that possess compounds rich in antioxidative properties (Table 2).

There are 4 types of antioxidants based on the defense mechanism.

- Preventive antioxidants: these suppress the free radical formation (i.e., enzymes such as peroxidase, catalase, lactoferrin, carotenoids, etc.).
- Radical scavenging antioxidants: these suppress the chain initiation reaction (i.e., vitamin C and carotenoids).
- Repair and *de novo* antioxidants: these comprise of proteolytic enzymes and repair enzymes for DNA and genetic materials.
- Enzyme inhibitor antioxidants: these induce the production and reaction of free radicals and the transport of the appropriate antioxidants to the appropriate active site.

The basic science that underlies the role of free radicals in causing cellular pathologies and the role of antioxidants in preventing this show that an imbalance between the ROS and antioxidant defense systems may lead to chemical modifications of biologically relevant macromolecules. This imbalance provides a logical pathobiochemical mechanism for the initiation and development of elderly anemia. Experimental data obtained *in vivo* provide evidence that antioxidants function in systems that scavenge reactive oxygen species and that these are relevant to what occurs *in vivo*. 

The majority of the antioxidant activity is due to the flavones, isoflavones, flavonoids, anthocyanin, coumarins, catechins, and isocatechins. Spices and herbs are recognized as sources of natural antioxidants and thus play an important role in the chemoprevention of diseases and aging.

*Rhodiola rosea* is commonly used in China and Tibet folk medicine for the treatment of high altitude sickness, anoxia, and mountain hypoxia. Salidroside (SDS) is an active ingredient of Rhodiola rosea. A recent study attempted to examine the potential erythropoiesis-stimulating and antioxidative effect of SDS in TF-1 erythroblasts. The erythropoiesis-promoting effect was determined by treating human TF-1 cells, a popular *in vitro* model for studying erythropoiesis, with SDS in the presence and absence of EPO through the measurement of the expression of a series of erythroid markers such as glycophorin A (GPA), transferrin receptor (CD71), and hemoglobin (Hb). The potential protective effect of SDS against H_2_O_2_-induced apoptosis and its underlying mechanism in TF-1 erythroblasts were examined by flow cytometry and Western blot analysis. SDS promotes erythropoiesis in the EPO-treated cells and it also reduces the number of apoptotic cells in TF-1 erythroblasts after H_2_O_2_ treatment, most likely through the upregulation of the protective proteins thioredoxin-1 (Trx1) and glutathione peroxidase-1 (GPx1). These findings support the use of SDS as an erythropoiesis-adjuvant agent to correct anemia and hypoxia [112].

*Spirulina* is filamentous and multicellular blue-green algae capable of reducing inflammation and manifesting antioxidant effects. Selmi et al. [113] hypothesized that *Spirulina* may ameliorate anemia and immunosenescence in the elderly with a history of anemia. They enrolled 40 volunteers of both sexes, age of 50 years or older, who had no history of major chronic diseases. Participants took a *Spirulina* supplementation for 12 weeks and were administered comprehensive dietary questionnaires to determine their nutritional regimen during the study. Complete cell count and indoleamine 2, 3-dioxygenase (IDO) enzyme activity, as a sign of immune function, were determined at baseline and weeks 6 and 12 of supplementation. Over the 12-week study period, there was a steady increase in the average values of the mean corpuscular hemoglobin in subjects of both sexes. In addition, the mean corpuscular volume and mean corpuscular hemoglobin concentration also increased in male participants. Older women appeared to benefit more rapidly from Spirulina supplements. Similarly, the majority of subjects had an increased IDO activity and white blood cell count at 6 and 12 weeks of Spirulina supplementation. Therefore, Spirulina may ameliorate anemia and immunosenescence in older subjects. Large human studies to determine whether this safe supplement could be beneficial in randomized clinical trials are warranted.

Among antioxidants, oral N-acetylcysteine (NAC) supplementation was tested for the treatment of anemia and oxidative stress in hemodialysis (HD) patients [114]. Forty-nine hemodialysed patients received NAC 200 mg orally thrice a day during the first 3 months, while 276 patients not receiving NAC were observed. The demographic and laboratory data of both groups were similar at baseline. When the erythropoietin dosage was stable throughout, only the NAC group had a significant increase in hematocrit, accompanied with a decrease in plasma levels of 8-isoprostane and oxidized low-density lipoprotein. Analyzed as a nested case-control study, NAC supplementation was also found to be a significant predictor of positive outcomes in uremic anemia.

*Curcuma longa* (turmeric) has a long history of use in Ayurvedic medicine as a treatment for inflammatory conditions. Turmeric constituents include the following three curcuminoids: curcumin (diferuloylmethane, the primary constituent and the one responsible for its vibrant yellow color), demethoxycurcumin, and bisdemethoxycurcumin, as well as volatile oils (tumerone, atlantone, and zingiberene), sugars, proteins, and resins. Among nutraceuticals, the role of *curcumin* is supported by a number of scientific papers that have confirmed its anti-inflammatory and antioxidant actions both *in vitro* and *in vivo*. *Curcumin* is the phytochemical derived from the rhizome of Curcuma longa, present in the spice tumeric that gives Indian curry its yellow color. *Curcumin* has been used for millennia as a wound-healing agent and to treat a variety of diseases in traditional Indian and Chinese medicine. *Curcumin* has an unprecedented number of molecular targets justifying its antioxidant and anti-inflammatory activities. Briefly, these targets include transcription factors with AP-1 and others such as SP-1, p53, STAT-3, AFT3, Nrf2, PPAR-*γ*, CHOP, HIF-1*α*, *β*-catenin, and NF-*κ*B, enzymes such as protein kinases (PKA, PKC, FAk, Src), glutathione S-transferase, DNA topoisomerase-II, telomerase, heme-oxygenase-1, p300 histone acetyltransferase, metalloproteinase, lipoxygenase (5-LOX), cyclooxygenase-2 (COX-2), and others. The most far-reaching physiological consequences seem to stem from the action of curcumin as an inhibitor of the activity of the transcription factor NF-*κ*B. The NF-*κ*B transcription factor is a master regulator of the inflammatory process, which activates the expression of many pro-inflammatory cytokines, such as TNF-*α*, IL-1*β*, and IL-6. Some of the NF-*κ*B-induced proteins, like TNF-*α*, are also its activators, which is particularly important in a chronic inflammatory state. NF-*κ*B seems to be the culprit of inflammaging because this signaling system integrates the intracellular regulation of immune responses in both aging and age-related diseases. Many activities of curcumin can also be explained by its ability to suppress acute and chronic inflammation by scavenging reactive oxygen and reactive nitrogen species and enhancing antioxidant defense (i.e., by increasing the glutathione level). Curcumin is not only a simple antioxidant but also an electrophilic compound that triggers the Nrf2/ARE signaling pathway which plays a key role in activating antioxidative enzymes, phase 2 enzymes, and so-called vitagenes (heme oxygenase, Hsp70, thioredoxin reductase, and sirtuins) that might have a pivotal role in oxidative stress-induced diseases. The amount of data documenting the protective effects of curcumin for different diseases, particularly those related to age, is increasing. It seems that the extraordinary potency of curcumin, which makes it an almost universal remedy, results from the inflammatory origins of many diseases and curcumin's anti-inflammatory activity. Despite the practical lack of data showing curcumin's influence on aging and lifespan, there is a strong rational argument suggesting that curcumin can influence the process of senescence and ageing retardation. Curcumin has numerous pharmacological activities, including antioxidant, antimicrobial, and anti-inflammatory properties. Research has proven curcumin to be a highly pleiotropic molecule capable of interacting with numerous molecular targets involved in inflammation. Based on early cell culture and animal research, clinical trials indicate curcumin may potentially be a therapeutic agent for inflammatory diseases. Because of curcumin's rapid plasma clearance, and conjugation, its therapeutic usefulness has been somewhat limited, leading researchers to investigate the benefits of curcumin complexes with other substances to increase the systemic bioavailability. Numerous in-progress clinical trials should provide an even deeper understanding of the mechanisms and therapeutic potential of curcumin [115].

 The use of complementary medicines, such as plant extracts, in therapy varies according to the different cultural traditions. However, the antioxidant and neuroprotective actions of some phytochemicals and plant extracts, such as gingko biloba, have been demonstrated in the treatment and prevention of dementia pathologies, particularly Alzheimer's disease [116]. Also fermented papaya preparation (FPP) (a product of yeast fermentation of *Carica papaya* Linn) has been tested in chronic and degenerative disease conditions (such as thalassemia, cirrhosis, diabetes, and aging) and performance sports showing the ability to favorably modulate immunological, hematological, inflammatory, vascular, and oxidative stress damage parameters. Neuroprotective potential evaluated in an Alzheimer's disease cell model showed that the toxicity of the *β*-amyloid could be significantly modulated by FPP. FPP modulated the oxidative stress-induced apoptosis through the downregulation of the ERK, Akt, and p38 activation mediated by H_2_O_2_. FPP reduces the extent of the H_2_O_2_-induced DNA damage, an outcome corroborated by similar effects obtained in the benzo[a]pyrene treated cells [117].

 Other plants and their extracts that have produced promising clinical data in dementia patients, with respect to cognition, include saffron (*Crocus sativus*), *ginseng* (*Panax* species), sage (*Salvia* species) and lemon balm (*Melissa officinalis*), although more extensive and reliable clinical data are required [118].

 Since oxidative stress-induced cell damage and inflammation are implicated in the pathogenesis of anemia of chronic inflammation, this condition could potentially benefit from functional nutraceutical/food supplements exhibiting anti-inflammatory, antioxidant, and immunostimulatory and antioxidant properties.

## 6. Conclusions 

With the continued elderly population growth, anemia will likely continue to be a significant economic burden on society. The challenge for treating and laboratory-based physicians is to understand the underlying causes and contributing factors that result in anemia in the elderly so that the potential value of emerging and innovative pharmacologic management can be considered. Including CRP and hepcidin in the diagnostic algorithm for anemia may better discriminate between classic iron-deficiency anemia (low hepcidin levels) and iron-deficiency anemia in the context of anaemia of inflammation or chronic disease (elevated hepcidin levels) [119]. 

The erythropoietic agents have the potential to play a therapeutic role in this patient population. Though the use of ESAs have negative consequences (e.g., increased blood pressure and thromboembolism), the risks of Epo therapy should be weighed against the potential beneficial effects of improving anemia, quality of life, and neurocognitive performance and decreasing the impact of ischemia in the brain, heart, and other organs. The molecular basis of anemia in a significant number of elderly people remains to be discovered. When this basis has been discovered, we propose that a rational, targeted therapy of the pathophysiological mechanism(s) of anemia should be more effective and likely safer than a nonspecific stimulation of erythropoiesis [120]. Accordingly, IV iron therapy associated with ESAs, emerging hepcidin antagonists, and other emerging ESA agents will each provide important new tools for pharmacologic alternatives in the management of anemia of inflammation in the elderly.

Considering the etiopathogenetic mechanisms of anemia of inflammation in the elderly population, an integrated nutritional/dietetic approach with nutraceuticals that can manipulate oxidative stress and related inflammation may prevent the onset of this anemia and its negative impact on patients' performance and quality of life. Indeed, emerging evidence suggests that interventions, including nutrition, pharmacology, and physical exercise, may activate the expression of cellular antioxidant systems and play a role in preventing inflammatory processes. For these reasons, new effective interventions, based on nutrition and aimed at targeting oxidative stress and chronic inflammation, may induce an important protection. Although a large body of research has focused on individual or small numbers of antioxidants, increasing the circulating antioxidant capacity through the increased consumption of antioxidant-rich fruits and vegetables may be protective against aging-related disease. The available scientific evidence indicates that the link between oxidative stress, a proinflammatory systemic environment, and aging anemia is strong. As previously demonstrated, anemia in elderly is associated with a low-grade inflammatory status, and a potent regulation of inflammatory mediators may not be required consequently. Contrarily, a slight or moderate inhibition of inflammation with plant extracts and phytonutrients, such as those used in the traditional medicines, might be useful to prevent the development of anemia and lead toward an increased longevity and enhanced quality of life [121].

## Figures and Tables

**Table 1:** Different options of treatment of anemia of inflammation in the elderly.

Treatment with clinical evidence	Rationale	Benefits/risks
Erythropoietic stimulating agents (ESAs)	EPO deficiency	Hb increase, fatigue reduction, QL improvement/thrombotic events, decreased survival
Oral iron	Iron deficiency anemia (ferritin < 30 ng/mL)	Hb increase/poor intestinal absorption, gastrointestinal side effects, poor compliance
IV Iron supplementation	Anemia of inflammation with iron functional deficiency	Improvement of Hb response to ESAs/hospitalization, infusion reaction
Lactoferrin	Anemia of inflammation with iron functional deficiency	Improvement of Hb response to ESAs Modulation of iron distribution Reduction of inflammation/no severe side effects

Emerging drugs	Rationale	Evidence

Hepcidin antagonists	Hepcidin-mediated iron restriction in anemia of inflammation	Preclinical studies
Anti IL-6 monoclonal Ab	Anemia of inflammation	One phase II clinical trial
Activators of autophagy (rapamycin)	Anemia of inflammation	Preclinical studies
AMPK activators (metformin, curcumin, etc.)	Anemia of inflammation	Preclinical studies
Nutraceuticals (spirulina, curcumin, ginkgo biloba, ginseng, fermented papaya)	Modulation of oxidative stress and inflammation	Preclinical and phase II clinical trials

**Table 2:** Categories of antioxidants.

(1) Endogenous antioxidants	(a) Enzymatic
	(b) Nonenzymatic

(2) Exogenous antioxidants	(a) From natural sources (i.e., secondary products of plants which are functioning as antioxidants):
	(i) Chlorophyll derivatives
	(ii) Essential oils
	(iii) Carotenoids
	(iv) Alkaloids
	(v) Phytosterols
	(vi) Phenols: coumarins, flavonoids
	(vii) Polyphenols: tannins, proanthocyanidins
	(viii) Nitrogen-containing compounds: alkaloids, indoles
	(b) Dietary antioxidants
